# Supplementary material for: Histone and Histone Acetylation-Related Alterations of Gene Expression in Uninvolved Psoriatic Skin and Their Effects on Cell Proliferation, Differentiation, and Immune Responses
Source: Int J Mol Sci. 2023 Sep 26;24(19):14551. doi: 10.3390/ijms241914551 (PMC10572426; doi:10.3390/ijms241914551)
Supplement: Supplementary file 1 [file ijms-24-14551-s001.zip › supplementary figure S1.pdf]

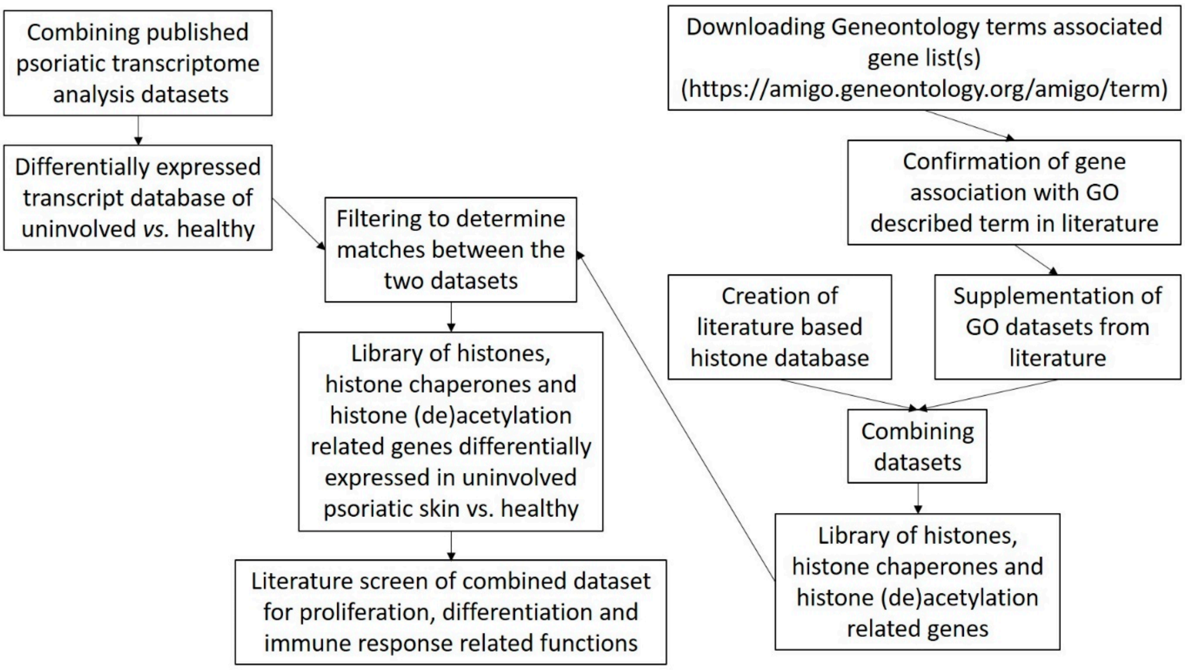

Supplementary Figure S1. The strategy of literature mining to confirm GO term association of genes in literature, and to identify function(s) of GO terms associated and literature supplemented genes was performed as described previously (ref). In brief, to identify function(s) of differentially expressed transcripts (uninvolved vs. healthy skin) associated with literature supplemented GO term (listed below) datasets (supplementary table 1.) literature mining was carried out using the HGNC (HUGO Gene Nomenclature Committee) gene symbol(s) applying the following strategy: after downloading the GO term associated gene lists (Human, https://amigo.geneontology.org/amigo/term) by applying Geneontology ID numbers (listed below), first each downloaded gene name was searched together with a given GO term as keywords to confirm GO described association in literature using PubMed (https://pubmed.ncbi.nlm.nih.gov/) online databases. Output publications were checked manually within the manuscript, as well as in Genecards human gene database (https://www.genecards.org) to confirm association of each gene with the GO term described mechanism.

To supplement downloaded GO datasets with additional associated genes from literature, Data of the AMIGO database was compared with the PubMed (https://pubmed.ncbi.nlm.nih.gov/) literature database using keyword-based automated literature screen. Output publications were checked manually within the manuscript, as well as in Genecards database for additional genes related to the given GO term described process/mechanism. If AMIGO database was found to be incomplete after a manual check based on the literature data, we combined the two results, and included the literature-supported relevant data. These literary additions are listed in a separate tab in Supplementary Table 1, together with the corresponding AMIGO databases.

In the case of histones, the AMIGO GO term “Histone(s)” was not available, so after a keyword-based automated literature screen, we created the histone database based on literature data. This was based on an article containing the classification of histones, which, to the best of our knowledge, contains all currently known human histone variants. (Stefano Amatori et al. The dark side of histones: genomic organization and role of oncohistones in cancer; 2021.).

These datasets were then combined given rise to the library of histones, histone chaperones and histone (de)acetylation related genes. This library and the transcriptome database of

differentially expressed transcripts of uninvolved psoriatic vs. healthy skin, (created by combining three published transcriptome analysis datasets,) was filtered to determine matches between the two datasets. The output of this filtering was a library of histones, histone chaperones and histone (de)acetylation related genes differentially expressed in uninvolved psoriatic skin vs. healthy. This library served as an input for a third literature screen together with given mechanism(s) term(s) (listed below) as keyword(s) to identify proliferation, differentiation and immune response related functions of (gene) dataset components. For this screen in PubMed the following keywords (listed in alphabetical order) were applied: Cell cycle, Dendritic cell, Differentiation, Epidermis, Immune, Immune cell, Innate immune, Inflammation, Hematopoietic and Hematopoiesis, Keratinocyte, Macrophage, Neutrophil, Pluripotency, Proliferation, Psoriasis, Self-renewal, Senescence, Skin, Stem cell, T cell.
All described screens and filtering(s) were conducted in a case insensitive manner (both upper- and lower-case letters were considered) and alternative names/aliases of genes were taken into account.

1. Histone chaperone activity, Geneontology ID#: GO:0140713

   Database used for literature search: Pubmed (https://pubmed.ncbi.nlm.nih.gov/)

   Keywords used for literature search to supplement Geneontology dataset: Histone chaperone, Histone chaperone complex

   Applied literature for supplementation: Assala Lamaa et al. Integrated analysis of H2A.Z isoforms function reveals a complex interplay in gene regulation, 2020; Dan Filipescu Developmental et al. roles of histone H3 variants and their chaperones, 2013; Daniel Moreno-Andrés et al. VPS72/YL1-Mediated H2A.Z Deposition Is Required for Nuclear Reassembly after Mitosis, 2020; Leanne De Koning et al. Histone chaperones: an escort network regulating histone traffic, 2007

2. Histone acetylation, Geneontology ID#: GO:0016573

   Database used for literature search: Pubmed (https://pubmed.ncbi.nlm.nih.gov/)

   Keywords used for literature search to supplement Geneontology dataset: Histone acetylation, Histone acetyltransferase, Histone acetyltransferase complex

   Applied literature for supplementation: Dominik A et al. Herbst Structure of the human SAGA coactivator complex, 2021; Liliana Arede et al. Buffering noise: KAT2A modular contributions to stabilization of transcription and cell identity in cancer and development 2021; Qilian Yang et al. Epigenetics in ovarian cancer: premise, properties, and perspectives, 2018; Sang-beom Seo et al. Regulation of Histone Acetylation and Transcription by Nuclear Protein pp32, a Subunit of the INHAT Complex, 2002; Vincenzo Di Cerbo et al. Cancers with wrong HATs: the impact of acetylation, 2013, Zhi Fang et al. The Role of Histone Protein Acetylation in Regulating Endothelial Function, 2021,

3. Histone deacetylation, Geneontology ID#: GO:0016575

   Database used for literature search: Pubmed (https://pubmed.ncbi.nlm.nih.gov/)

Keywords used for literature search to supplement Geneontology dataset: Histone deacetylase, Histone deacetylase complex, Histone deacetylation

Applied literature for supplementation: Epigenetics in ovarian cancer: premise, properties, and perspectives, 2018; Zhi Fang et al. The Role of Histone Protein Acetylation in Regulating Endothelial Function, 2021. Qilian Yang
